# SIMS Investigation of Al Diffusion Across Interfaces in AlGaN/GaN and AlN/GaN Heterostructures

**DOI:** 10.3390/nano16020125

**Published:** 2026-01-17

**Authors:** Jihed Laifi, Mohamed Fathy Hasaneen, Amor Bchetnia

**Affiliations:** 1 Department of Physics, College of Science, Jouf University, Sakaka P.O. Box 2014, Saudi Arabia; mfahmad@ju.edu.sa; 2 Department of Physics, College of Science, Qassim University, Buraydah Almolaydah, P.O. Box 6644, Buraydah 51452, Saudi Arabia; o.bchetnia@qu.edu.sa

**Keywords:** diffusion, SIMS, AlGaN, thermal annealing

## Abstract

This study investigates Metal–Organic Vapor Phase Epitaxy (MOVPE)-grown AlGaN/GaN and AlN/GaN heterostructures using high-temperature thermal annealing and Secondary Ion Mass Spectrometry (SIMS). By fitting experimental diffusion coefficients (DAl) to the Arrhenius equation, two crucial kinetic parameters were found: the activation energy (E_a_) and the pre-factor (D_0_). In the AlGaN/GaN structure, the dominating out-diffusion of Al has a large D_0_ = 4.03 × 10^−5^ cm^2^ s^−1^ and a low activation energy in the range of [2.1–2.4 eV]. A substitutional diffusion mechanism in the crystal lattice mediated by defects is closely linked to the low E_a_. Significantly higher activation energies (E_a_) of 3.66 and 4.59 eV, respectively, control both in- and out-diffusion processes in the AlN/GaN structure. The better intrinsic thermal stability of the pure AlN layer, whose stability is attained by a strong energy barrier, is confirmed by the increase of more than 1.2 eV in E_a_.

## 1. Introduction

High Electron Mobility Transistors (HEMTs) and other next-generation high-power and high-frequency electronic devices are built on the rapidly developing wide-bandgap semiconductors, especially gallium nitride, GaN [1,2,3]. GaN-based HEMTs rely heavily on the generation of a two-dimensional electron gas (2DEG) at the interface of the GaN channel and a high-bandgap barrier layer, which is commonly AlGaN or AlN. The performance and, more importantly, long-term reliability of these devices are inextricably tied to the thermal stability of this interface.

It is widely recognized that interface characteristics have a substantial impact on electronic device performance. As a result, research has concentrated on both thin film interface diffusion and film–substrate diffusion. Cai et al. [4] investigated the diffusion depth within AlGaN/GaN superlattices and proposed a simple method to reduce the abruptness of the AlGaN/GaN interfaces. They detected a significant asymmetry in Al diffusion depth between the upper (0.62 nm) and lower (0.96 nm) AlGaN contact borders. In addition, Li and Zhu [5] demonstrated that Al diffused from the Al_2_O_3_ substrate into the buffer layer during the growth of single-crystal GaN films, resulting in a buffer layer containing both GaN and AlN, despite the fact that Al was not added intentionally during the growth process. Furthermore, Haneda et al. [6] used a simplified error function model to investigate the Al ion diffusion patterns at the substrate/GaN thin film interface and concluded that the Al concentration within the films was higher than predicted by volume diffusion. Focusing on the GaN/sapphire interface, Kang et al. [7] determined the interdiffusion coefficients at 850 °C from SIMS profiles to be D_Al_ = 3.98 × 10^−17^ cm^2^ s^−1^ and D_Ga_ = 4.81 × 10^−17^ cm^2^ s^−1^. Furthermore, Fung et al. [8] studied the distribution of Ga and Al near the n-GaN/sapphire interface using X-ray energy-dispersive spectroscopy (XRDS) on a cross-section of the interface plane of n-GaN produced on sapphire using MOCVD. Their experimental results showed that Ga diffused further away from the sapphire substrate, whereas Al diffused less. In a recent work, we revealed that Al diffusion at the GaN/Al_2_O_3_ interface has an activation energy of 2.8 eV, implying that the process is dominated by a native defect-assisted substitutional diffusion mechanism rather than pure interstitial movement, whereas the interdiffusion of oxygen from the sapphire substrate into the GaN layer was found to have a substantially lower activation energy of approximately 1.24 eV, which we attributed to an interstitial migration mechanism [9].

Based on our previous study of Al diffusion during the growth of AlGaN/GaN and AlN/GaN heterostructures, in which we confirmed Al tailing into GaN layers and calculated the Al diffusion coefficients D_Al_ under Metal–Organic Vapor Phase Epitaxy (MOVPE) conditions [10], we now investigate further into the fundamental mechanism governing this Al migration. The initial work highlighted that D_Al_ is low (10^−14^–10^−15^ cm^2^ s^−1^ at 1100 °C) and strongly influenced by the interface quality and defect density, with diffusion being one order of magnitude higher at the highly mismatched AlN/GaN interface compared to AlGaN/GaN (x < 4). However, the simultaneous nature of high-temperature growth and diffusion makes it difficult to precisely isolate the thermodynamic parameters governing Al transport. To separate the diffusion process from growth kinetics and precisely define the controlling mechanism, this study used a systematic thermal annealing treatment of samples. We want to precisely determine the Al diffusion activation energy (E_a_) by identifying changes in the Al depth profiles during post-growth annealing at various temperatures. This critical parameter, combined with the slow diffusion rate, will allow us to definitively confirm whether Al diffusion in GaN is mediated. This complete technique is critical for attaining the required control over Al distribution at nitride interfaces to construct high-performance electrical and optoelectronic devices.

The effectiveness of two widely utilized barrier materials, a composite AlGaN layer and a pure AlN layer, is directly compared in the current study. We obtained high-resolution compositional depth profiles following high-temperature thermal annealing using Secondary Ion Mass Spectrometry (SIMS) to measure the thermal stability of these interfaces. The basic activation energies (E_a_) for Al diffusion were then computed using the Arrhenius equation after the experimental findings were analyzed using Fick’s diffusion laws to achieve the diffusion coefficients D_Al_. The aim of this work is to determine the activation energies (E_a_) and pre-factors (D_0_), which will provide the fundamental kinetic parameters needed for optimizing thermally stable GaN device structures, and to quantitatively compare the aluminum diffusion coefficients D_Al_ across the interfaces of the AlGaN/GaN and AlN/GaN heterostructures during thermal annealing using SIMS depth profiling.

## 2. Materials and Methods

**Figure 1:**
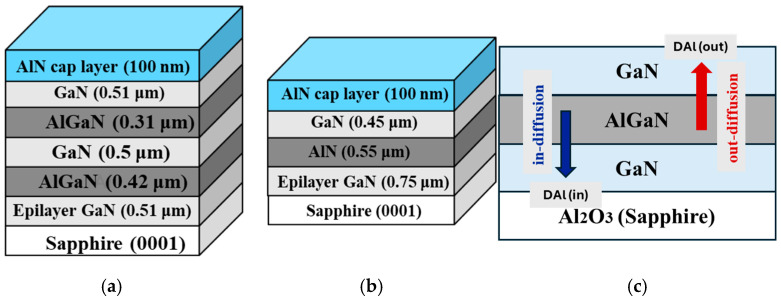
Schematic diagrams of GaN heterostructures grown on sapphire substrates: (**a**) AlN (cap)/GaN/2 × (Al_x_Ga_1−x_N/GaN), and (**b**) AlN (cap)/GaN/AlN/GaN. (**c**) Schematic diagram illustrating the in-diffusion and out-diffusion mechanisms in the AlGaN/GaN/sapphire heterostructure. The blue and red arrows represent the direction of in-diffusion (towards the substrate) and out-diffusion (towards the surface), respectively.

AlN (cap)/GaN/2 × (Al_x_Ga_1−x_N/GaN) and AlN (cap)/GaN/AlN/GaN heterostructures were grown on a c-plane (0001) sapphire substrate using Metal–Organic Vapor Phase Epitaxy (MOVPE) at atmospheric pressure. Ammonia (NH_3_), trimethylaluminum (TMAl), and trimethylgallium (TMGa) were used as N, Al, and Ga sources, respectively. Figure 1 shows the schematic representation of the two samples structures (A: AlN (cap)/GaN/2 × (Al_x_Ga_1−x_N/GaN)) and (B: AlN (cap)/GaN/AlN/GaN). More details on the growth process can be found in Ref. [11]. Four pieces were cut from the as-grown samples and labeled, respectively, as A_0_, A_1_, A_2_, and A_3_ for structure A, and B_0_, B_1_, B_2_, and B_3_ for structure B. The A_0_ and B_0_ samples were taken as references (as-grown samples); the other samples A_1–3_ and B_1–3_ were annealed for 25 min under N_2_ atmosphere at 1050 °C, 1100 °C, and 1150 °C, respectively. Then, in situ reflectometry measurements were used to investigate the thermal stability of the samples. Secondary Ion Mass Spectrometry (SIMS) measurements were carried out. The compositional depth profiles were obtained using a focused beam (Cs^+^) primary ion with an energy of 3 keV to monitor the elemental concentration Al versus depth.

## 3. Results

**Figure 2:**
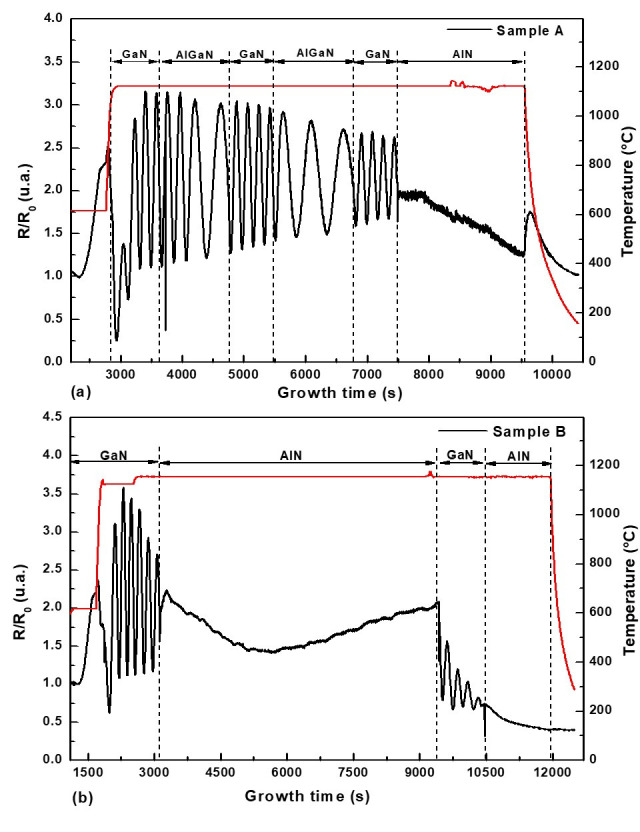
In situ reflectivity (black line) and growth temperature (red line) as a function of real time during the deposition of (**a**) AlN (cap)/GaN/2 × (Al_x_Ga_1−x_N/GaN) and (**b**) AlN (cap)/GaN/AlN/GaN.

The growth process of both Sample A (AlGaN/GaN multi-layer structure) and Sample B (GaN/AlN structure) was monitored in situ using laser reflectivity. Figure 2 shows the normalized reflectivity signals (R/R_0_) for the two different structures tested in this work. The reflectivity trace of Sample A (Figure 2a) shows distinct oscillations, indicating a steady, two-dimensional (2D) growth mode with smooth surfaces. The period and amplitude change as the GaN layers transition to the AlGaN barrier layers. These changes are consistent with the modification of the material’s refractive index and the change in the growth rate of the AlGaN composition when compared to GaN. The persistence of these distinct oscillations during the different phases of GaN/AlGaN suggests a highly stable and reproducible growth mode, which is critical for realizing the multi-layer heterostructure illustrated in Figure 1. The reflectivity curve of Sample B (Figure 2b), which corresponds to the structure with a single, pure AlN barrier, similarly shows well-defined oscillations in the first GaN layer, suggesting an initial 2D development phase. However, after the thick AlN barrier is initiated, the oscillations dampen dramatically and become nearly flat. This substantial dampening and subsequent signal stabilization are expected for the formation of a thick AlN layer, implying an increase in surface roughness or a shift to a three-dimensional (3D) growth component. The abrupt return of clear, high-amplitude oscillations following the addition of the final GaN cap layer demonstrates the technique’s sensitivity to the material being deposited and implies that the GaN can easily smooth the underlying AlN surface.

**Figure 3:**
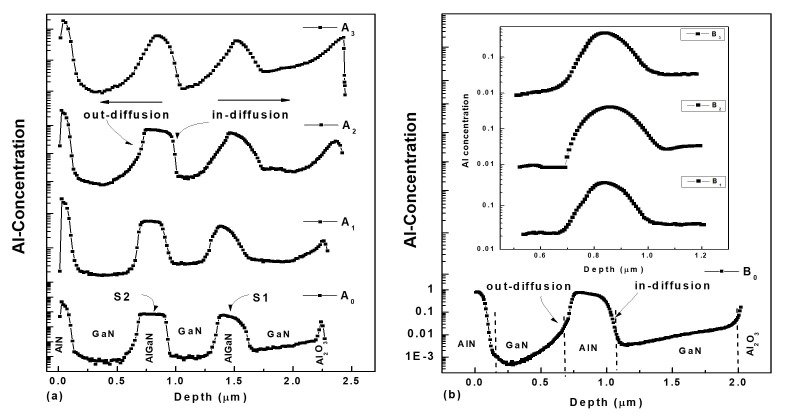
(**a**) SIMS analysis of Al concentration of the as-grown A_0_ sample and upon thermal annealing at different temperatures (A_1_–A_3_). (**b**) SIMS analysis of Al concentration of the as-grown B_0_ sample and upon thermal annealing at different temperatures (B_1_–B_3_).

Figure 3a,b depicts the SIMS profiles of as-grown and annealed samples for both A and B structures. The results show how the thermal annealing temperature affects the concentration of aluminum (Al) within the heterostructures as a function of the depth. As shown in Figure 3a, Sample A_0_ (as-grown) serves as the reference, with abrupt interfaces and a nearly square-wave profile for Al intensity, which are typical of the initial, well-defined AlN cap, GaN spacers, and AlGaN interlayers (S1 and S2). However, for the annealed samples (A_1_, A_2_, and A_3_) at higher temperatures (1050 °C to 1150 °C), the Al SIMS profile gradually broadens, demonstrating a clear relationship between increased thermal energy and Al diffusion. This diffusion is characterized by two processes, as shown in Sample A_2_. The terms out-diffusion and in-diffusion are defined by the direction of aluminum (Al) migration relative to the intentionally grown barrier layer. Out-diffusion refers to Al atoms migrating from the barrier layer toward the top surface of the sample. This occurs along the c-axis [0001] direction of the GaN crystal lattice. In-diffusion refers to Al atoms moving from the barrier layer toward the sapphire substrate. This occurs along the -c-axis [000-1] direction. The two sandwiched AlGaN spikes (S1 and S2) were identified. We found 1.9% and 2.3% of Al solid composition in S1 and S2, respectively. The Al solid composition of the AlGaN layer (the peak intensity) remains practically constant, implying that the diffusion mechanism impacts the interfacial areas rather than causing Al redistribution throughout the interlayer volume. The SIMS depth profiles (Figure 3b) for the AlN/GaN structure (Sample B), which has a single AlN interlayer, clearly indicate a thermally activated diffusion process, resulting in significant interface broadening and a significant decrease in the AlN peak concentration (from B_0_ to B_3_). The higher loss of Al from the layer when compared to the AlGaN structure is due to the pure AlN (x = 1) acting as a more concentrated Al source.

For a quantitative investigation, the SIMS depth profiles can be modeled using a solution to Fick’s second law, described by the following equation [10]:

(1) $$C\left(x,t\right)=C_{0}\;\exp(\frac{{-\left(x-x_{0}\right)}^{2}}{4Dt})$$

where C is the Al concentration at a given depth x after annealing time t, C_0_ is the initial concentration, x_0_ is the interface position, and D is the diffusion coefficient.

**Figure 4:**
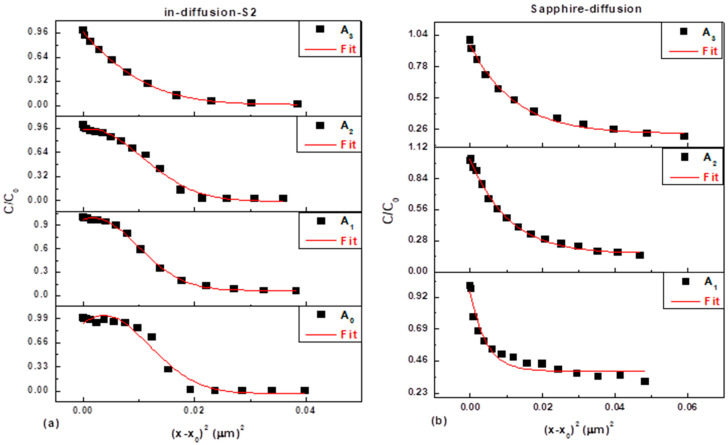
(**a**) Al SIMS depth profiles for the different AlGaN/GaN interfaces for Sample A before and after thermal annealing. (**b**) Al SIMS depth profiles for the different GaN/Al_2_O_3_ interfaces for Sample A after thermal annealing. Black lines represent the experimental Al SIMS data. The red line represents the best fit of the profile broadening using the Gaussian function model.

The increase in broadening observed in the profiles A_1_–A_3_ and B_1_–B_3_ suggests a direct increase in the Al diffusion coefficient (D_Al_) as the thermal annealing temperature increases. This temperature dependence is crucial for understanding and controlling thermal stability at the AlGaN/GaN and AlN/GaN interfaces. To quantify this process, the SIMS data was successfully fitted using the Gaussian function solution to Fick’s second law, as shown in Figure 4, confirming that the diffusion process can be accurately modeled across various interfaces.

Table 1 summarizes the results of the Al diffusion coefficient for the in-diffusion (D_in_) and out-diffusion processes (D_out_) at the various AlGaN/GaN interfaces of sample (A).

The comprehensive fitting of the Al diffusion coefficients (D_Al_) across the two AlGaN/GaN interfaces, S1 and S2, indicates a highly complicated and asymmetric thermal instability. The dominant process at the inner AlGaN/GaN interface (S1) is in-diffusion (D_in-S1_), in which Al atoms migrate into the AlGaN layer at a rate up to ten times faster than out-diffusion at 1050 °C. The thermal diffusion results for D_in-S1_ show a near-constant behavior across the temperature range studied, with values decreasing slightly from 1.51 × 10^−14^ cm^2^ s^−1^ at 1050 °C to 1.17 × 10^−14^ cm^2^ s^−1^ at 1150 °C. This non-Arrhenius behavior indicates that the process is not simply thermally activated; it is probably dominated by a competing mechanism, such as stress relaxation or a temperature-driven change in native defect concentration that restricts Al mobility at higher temperatures. The inner interface out-diffusion (D_out-S1_) is slower but is thermally activated. Conversely, the AlGaN/GaN interface (S2), displays a clear and distinct behavior: out-diffusion (D_out-S2_) is the dominant mechanism, showing a rapid rate of between 2.01 × 10^−14^ cm^2^ s^−1^ and 9.50 × 10^−14^ cm^2^ s^−1^, with D_out-S2_ being up to 100 times larger than D_in-S2_. This dominant out-diffusion of Al from the barrier layer into the adjacent GaN layer is the primary cause of interface broadening. Our results reveal a distinct variation in diffusion kinetics between S1 and S2. This difference is attributed to the evolution of the GaN buffer morphology during growth. The first interface (S1) was deposited on a GaN layer prior to full coalescence, a phase where the material contains a higher concentration of grain boundaries and structural defects. According to our previous structural investigation of GaN growth stages, this near-interface region is characterized by a high threading dislocation (TD) density; the total density is estimated to be significantly higher than the values found in fully coalesced films. Specifically, the screw-type dislocation density in the early growth stages can reach 10^9^ cm^−2^, while edge-type dislocations are even more prevalent, at approximately 10^10^ cm^−2^ [12]. These defects act as primary sources for gallium vacancies (V_Ga_) and provide efficient pathways for the substitute migration of Al atoms. As growth proceeds to the second interface (S2), the GaN template reaches full coalescence and transitions to a 2D growth mode, leading to a substantial reduction in the density of these structural pathways. For fully coalesced layers, the dislocation density effectively drops by nearly an order of magnitude, with screw-type densities falling to 10^8^ cm^−2^. This reduction explains the observed stability and lower diffusion rates at the S2 interface compared to the S1 region. Furthermore, the enhanced Al diffusion toward the top AlN cap (D_out-S2_) compared to the limited diffusion toward the sapphire substrate (D_in-S2_) can be attributed to the strain gradient induced by the AlN cap. This strain gradient influences atomic diffusion by introducing a stress-assisted flux that enhances migration toward layers with larger lattice mismatch. The top AlN cap exerts a stronger compressive strain on the adjacent GaN layers compared to the strain regime near the sapphire substrate, thereby favoring upward Al migration in the second AlGaN layer (S2) [13].

**Figure 5:**
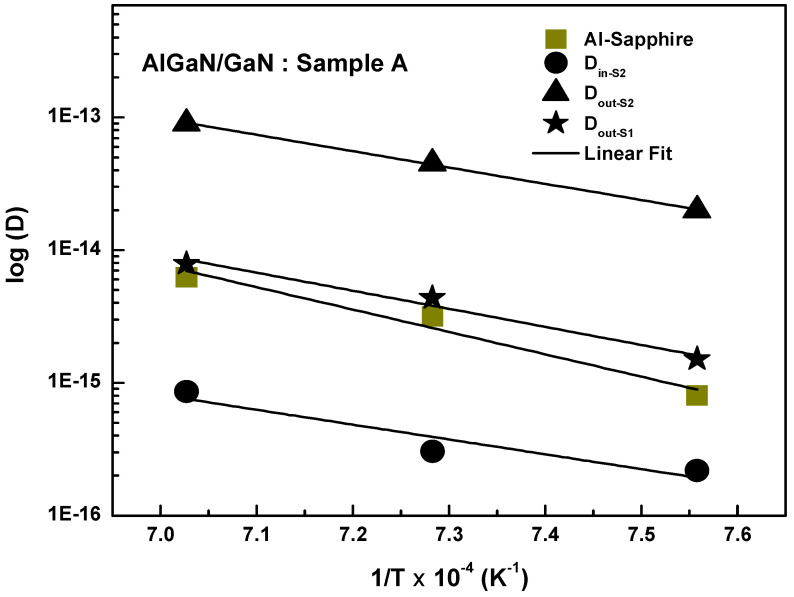
Arrhenius plot of the Al diffusion constant, D_out-S1_, D_in-S2_, D_out-S2_, and D_Al-sapphire_, for Sample A.

The diffusion determined from these fits was then analyzed using the Arrhenius relation, $D=D_{0}\exp\left(-\frac{E_{a}}{K_{B}T}\right)$, as shown in Figure 5, where D_0_ is a pre-factor, E_a_ is the activation energy, T is the annealing temperature, and K_B_ is the Boltzmann constant, confirming that all three processes, D_out-S1_, D_in-S2_ and D_out-S2_, are thermally activated.

The extracted activation energies (E_a_) and pre-factors (D_0_) provide the microscopic interpretation of the diffusion mechanism. The D_out-S2_ (out-diffusion from AlGaN) is characterized by the following equation: $D_{out\text{-}S2}=4.03\times 10^{-5}\;{\mathrm{cm}}^{2}s^{-1}\exp\left(-\frac{2.44\;eV}{K_{B}T}\right)$. The relatively low E_a_ = 2.44 eV suggests a defect-mediated mechanism, likely via Ga (V_Ga_) and N (V_N_) vacancies, which are easily formed in the strained AlGaN layer [14,15]. The high D_0_ = 4.03 × 10^−5^ cm^2^ s^−1^ (the largest at the S2 interface) is the rate-determining factor for Al out-diffusion, indicating a significantly higher concentration of (V_Ga_) defects within the AlGaN layer compared to the GaN layer. The D_in-S2_ (in-diffusion into AlGaN) is described by $D_{in\text{-}S2}=5.33\times 10^{-8}\;{\mathrm{cm}}^{2}s^{-1}\exp\left(-\frac{2.21\;eV}{K_{B}T}\right)$. The inner interface out-diffusion (D_out-S1_) follows the standard Arrhenius law: $D_{out\text{-}S1}=3.09\times 10^{-5}\;{\mathrm{cm}}^{2}s^{-1}\exp\left(-\frac{2.70\;eV}{K_{B}T}\right)$.

The activation energies E_a_ = 2.44 eV, E_a_ = 2.21 eV, and E_a_ = 2.70 eV are comparable and close to the 2.6 eV migration barrier reported for N vacancies in GaN [15], implying that the fundamental microscopic jump mechanism is a native defect-assisted substitutional diffusion. These low E_a_ values, when compared to the [3–5 eV] range for Al diffusion [9,15], are inconsistent with a simple interstitial mechanism and may thus be ruled out. Our findings support the basic principle that the local distribution and migration rate of native defects influence atomic behavior and diffusion rate. The quantitative analysis of the SIMS profiles indicates that aluminum (Al) diffusion occurs not only at the AlGaN/GaN interface, but also at the deeper GaN/sapphire interface, as illustrated in Figure 5. Diffusion at the substrate interface is defined by the following equation: $D_{Al\text{-}Sapphire}=4.85\times 10^{-3}\;{\mathrm{cm}}^{2}s^{-1}\exp\left(-\frac{3.34\;eV}{K_{B}T}\right)$. The quantitative analysis of Al diffusion from the sapphire (Al_2_O_3_) substrate into the GaN buffer layer yielded a diffusion coefficient of D_Al_ = 3.18 × 10^−15^ cm^2^ s^−1^ at 1100 °C and an activation energy (E_a_) of 3.34 eV. This value is precisely within the high [3–5 eV] range typically expected for Al diffusion in GaN, demonstrating that the process is highly thermally activated and must overcome a significant energy barrier. This high E_a_ significantly supports the claim that Al diffusion in GaN is slow and not controlled by a simple interstitial mechanism. Furthermore, our measured E_a_ of 3.34 eV is consistent with an investigation in the literature into Al diffusion from sapphire to GaN, which found an E_a_ of 3.35 eV [6]. This activation value is quite similar to the reported thermal migration barriers for GaN thermal degradation (3.2 eV), supporting the significance of native defects in Al migration. According to Deppe et al., the self-diffusion of column III lattice atoms in semiconductors has to proceed via native crystal defects, and the overall diffusivity is determined by the native defect migration rate and concentration [16]. Ono et al. observed this concept, reporting that the local distribution of vacancies in the crystal controls Al atomic behavior during diffusion [17]. Despite the consistency in the E_a_ (the energy barrier), our measured diffusion coefficient at 1100 °C is two orders of magnitude lower (D_Al_ = 3.18 × 10^−15^ cm^2^ s^−1^) than the (D_Al_ = 4.8 × 10^−14^ cm^2^ s^−1^) extrapolated from Haneda et al. [6]. The lower D_Al_ in our work indicates that the GaN/sapphire interface is less dislocated than the material examined by Haneda et al. Our estimated pre-factor (D_0_ = 4.85 × 10^−3^ cm^2^ s^−1^) for the GaN/sapphire interface, which is in fact two orders of magnitude greater than those of the AlGaN interfaces, reflects this. However, the activation energy (E_a_) also has a significant influence on the diffusion coefficient D_Al_. According to our findings, E_a_ is significantly greater for the sapphire interface (3.34 eV) than for the AlGaN/GaN interfaces (2.2–2.7 eV). The strong Al-O bonds in the sapphire (Al_2_O_3_) lattice, which need more thermal energy to break than the Al-N bonds in the strained AlGaN layer, are the cause of this high barrier. This larger energy barrier acts as the dominant limiting factor, resulting in a lower diffusion coefficient (D_Al_) despite the higher defect concentration at the sapphire interface.

Table 2 summarizes the results of Al diffusion coefficients for in-diffusion (D_in_) and out-diffusion processes (D_out_) from sample (B) AlN/GaN interfaces.

**Figure 6:**
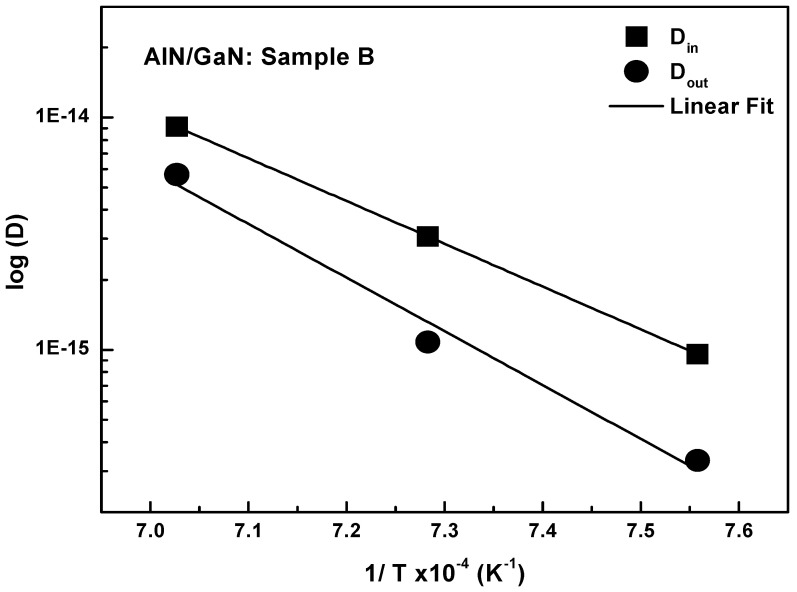
Arrhenius plot of the Al diffusion constant D_in_ and D_out_ for Sample B.

The diffusion coefficients for the annealed samples (B_1_–B_3_) are often lower than that for the as-grown sample (B_0_). This emphasizes the distinction between diffusion during MOVPE growth and during post-growth thermal annealing. Unlike the complex behavior seen at the AlGaN/GaN interfaces, D_in_ and D_out_ in the AlN/GaN structure increase monotonically when the annealing temperature rises from 1050 °C to 1150 °C. This temperature dependence implies that both diffusion processes are predominantly controlled by an Arrhenius-type thermally activated mechanism. The quantitative analysis of the structure (B), supported by the Arrhenius plot (Figure 6), reveals a shift in the kinetic control of the diffusion asymmetry, with D_in-AlN_ being more pronounced than D_out-AlN_.

The in-diffusion process (D_in-AlN_) is described by $D_{in\text{-}AlN}=8.11\times 10^{-2}\;{\mathrm{cm}}^{2}s^{-1}\exp\left(-\frac{3.66\;eV}{K_{B}T}\right)$. The out-diffusion process D_out-AlN_) is described by $D_{out\text{-}AlN}=1.09\times 10^{-2}\;{\mathrm{cm}}^{2}s^{-1}\exp\left(-\frac{4.59\;eV}{K_{B}T}\right)$. In this AlN/GaN structure, the E_a_ values (3.66 eV for D_in-AlN_ and 4.59 eV for D_out-AlN_) are significantly higher than those found in the AlGaN/GaN structure (2.2–2.4 eV), indicating a much more stable interface with a higher energy barrier required for Al diffusion. This enhanced stability is most likely due to the stronger binding strength and probably lower defect concentration in pure AlN compared to the AlGaN layer. The high E_a_ of 4.59 eV severely hinders the process of Al out-diffusion from the AlN layer, suggesting that the mechanism for Al escape from the concentrated AlN layer into the GaN requires overcoming a significant energy barrier, possibly due to a reduction in the required mobile defects at the boundary. In contrast, Al in-diffusion is promoted by a significantly lower E_a_ of 3.66 eV and a higher D_0_ (8.11 × 10^−2^ cm^2^ s^−1^), which is nearly eight times larger than the D_0_ for out-diffusion (1.09 × 10^−2^ cm^2^ s^−1^). The stress state at the bottom interface (S1, in-diffusion) differs from the top interface (S2, out-diffusion) as the layer relaxes. A higher activation energy for out-diffusion (E_a_ = 4.59 eV) indicates that the Al atoms are more bound in the AlN lattice near the surface, requiring more thermal energy to break their bonds and initiate migration. There is typically a higher density of dislocations near the first AlN/GaN interface compared to the top of the layer. These defects effectively lower the average energy barrier for Al atoms to jump, resulting in a lower E_a_ of 3.66 eV.

The strong asymmetry observed in the AlN/GaN structure is determined by the combination of a lower E_a_ and a greater D_0_, making Al in-diffusion the predominant route for interfacial mixing. This finding emphasizes that the kinetic control of Al diffusion is significantly altered by the particular composition (AlN vs. AlGaN) and the corresponding strain or defect distribution, necessitating careful attention when building thermally stable high-power electronic devices. Indeed, the AlN layer thickness significantly exceeds the critical thickness (~10 nm), resulting in a large density of misfit and threading dislocations as well as significant strain relaxation. In contrast to the ideal lattice, these defects offer high-diffusivity paths that promote Al migration. As a result, the mismatched AlN/GaN interface has a greater diffusion coefficient (D_Al_) than the AlGaN/GaN interface. Higher pre-factors (D_0_) further support this, indicating that dislocations serve as sources of gallium vacancies (V_Ga_), which raise the frequency of atomic jumps. Ultimately, while the Al-N bond strength imposes a high activation energy (E_a_), these growth-induced defects dominate the intermixing kinetics, explaining why AlN exhibits a higher D_0_ than the AlGaN layer. The improved intrinsic thermal stability of the pure AlN layer is confirmed by the significant rise in (E_a_), by more than 1.2 eV when compared to AlGaN, which is probably caused by its stronger Al-N bonds and distinct equilibrium defect concentration. Our findings verify that the AlN structure is maintained by a high energy barrier (E_a_), leading to noticeably better thermal stability for applications requiring high-temperature operation. These findings have important implications for the design of AlGaN/GaN HEMTs utilizing AlN interlayers. To improve 2DEG mobility, these interlayers must be kept thin enough to avoid reaching the critical relaxation threshold. Our data shows that once the layer relaxes and develops defects, these defects create highly diffusive pathways. In this state, the many high-speed pathways created by these defects (high D_0_) overcome the resistance of the strong chemical bonds, allowing atoms to migrate across the interface easily. Consequently, a relaxed AlN layer promotes faster intermixing across the interface. This causes interfacial intermixing and weakens the 2DEG confinement, which can compromise the device’s performance during high-temperature fabrication.

## 4. Conclusions

This study demonstrates that aluminum (Al) diffusion in both AlGaN/GaN and AlN/GaN heterostructures is a substitutional process mediated by native point defects. The AlGaN/GaN structure is characterized by a relatively low activation energy (E_a_) of 2.1–2.4 eV and a large pre-factor (D_0_ = 4.03 × 10^−5^ cm^2^ s^−1^), indicating lower thermal stability. Diffusion at these interfaces is highly asymmetric; out-diffusion is enhanced toward the top AlN cap due to a strain gradient that favors migration toward layers with larger lattice mismatch. The pure AlN/GaN interface exhibits significantly improved intrinsic thermal stability, with E_a_ values increasing by more than 1.2 eV compared to AlGaN. This high energy barrier (E_a_ = 3.66 eV for in-diffusion and 4.59 eV for out-diffusion) is attributed to stronger Al-N bonds and a higher energy requirement for atomic jumps. Al diffusion from the sapphire substrate into the GaN buffer yields an E_a_ of 3.34 eV. While defect-rich, this interface remains limited by the strong Al-O bonds in the sapphire lattice, which require more thermal energy to break than Al-N bonds in strained interlayers. Finally, the high-energy barrier provided by pure AlN layers is critical for maintaining interfacial integrity in high-performance, high-temperature GaN electronic devices.

## Figures and Tables

**Table 1:** Al diffusion coefficients (D_Al_) extracted from the Gaussian fits for the AlGaN/GaN heterostructure (Sample A).

Sample	T_ann_ (°C)	D_in-S1_ (cm^2^ s^−1^)	D_out-S1_ (cm^2^ s^−1^)	D_in-S2_ (cm^2^ s^−1^)	D_out-S2_ (cm^2^ s^−1^)
A_0_	As-Grown	1.35 × 10^−14^	1.46 × 10^−15^	7.73 × 10^−17^	1.26 × 10^−16^
A_1_	1050	1.51 × 10^−14^	1.51 × 10^−15^	2.18 × 10^−16^	2.01 × 10^−14^
A_2_	1100	1.43 × 10^−14^	4.37 × 10^−15^	3.05 × 10^−16^	4.52 × 10^−14^
A_3_	1150	1.17 × 10^−14^	7.93 × 10^−15^	8.62 × 10^−16^	9.50 × 10^−14^

**Table 2:** Al diffusion coefficients (D_Al_) extracted from the Gaussian fits for the AlN/GaN heterostructure (Sample B).

Sample	T_ann_ (°C)	D_in_ (cm^2^ s^−1^)	D_out_ (cm^2^ s^−1^)
B_0_	As-Grown	1.01 × 10^−14^	4.99 × 10^−15^
B_1_	1050	9.58 × 10^−16^	3.34 × 10^−16^
B_2_	1100	3.07 × 10^−15^	1.08 × 10^−15^
B_3_	1150	9.12 × 10^−15^	5.69 × 10^−15^

## Data Availability

All data generated or analyzed during this study are included in this published article.
